# Syndromic versus Laboratory Diagnosis of Sexually Transmitted Infections in Men in Moshi District of Tanzania

**DOI:** 10.1155/2020/7607834

**Published:** 2020-02-07

**Authors:** Yuwei Cheng, Elijah Paintsil, Musie Ghebremichael

**Affiliations:** ^1^Department of Mathematical and Computer Sciences, College of the Holy Cross, Worcester, MA, USA; ^2^Department of Pediatrics, Yale University School of Medicine, New Haven, CT, USA; ^3^Harvard Medical School, Ragon Institute of MGH, MIT and Harvard, Cambridge, MA, USA

## Abstract

The syndromic diagnosis of sexually transmitted infections (STIs) is widely recognized as the most practical, feasible, and cost-effective diagnostic tool in resource-limited settings. This study assessed the diagnostic accuracy of syndromic versus laboratory testing of STIs among 794 men randomly selected from the Moshi district of Tanzania. Participants were interviewed with a questionnaire that included questions on history of STIs symptoms. Blood and urine samples were taken from the participants for laboratory testing. Only 7.9% of the men reported any symptoms of STI; however, 46% of them tested positive for at least one STI. There was little agreement between syndromic and laboratory-confirmed diagnoses, with low sensitivity (0.4%–7.4%) and high specificity (96%–100%) observed for each individual symptom. The area under the receiver-operating curve was 0.528 (95% CI: 0.505–0.550), indicating that the syndromic approach has a 52.8% probability of correctly identifying STIs in study participants. In conclusion, whenever possible, laboratory diagnosis of STI should be favored over syndromic diagnosis.

## 1. Introduction

AIDS continues to be one of the leading causes of death in sub-Saharan Africa [1]. Among the 36.9 million people living with HIV globally_,_ 53% lived in sub-Saharan Africa [2]. Sexually transmitted infections (STIs) facilitate the transmission, disease progression, and treatment outcomes of HIV [3–5]. Moreover, people living with HIV (PLWH) have an increased prevalence of other STIs [6]. In sub-Saharan Africa, high incidence of untreated STIs has been associated with an increased rate of HIV transmission [7]. The World Health Organization (WHO) reported that other STIs such as syphilis and HSV-2 increase a person's risk of acquiring HIV infection by more than three-fold [8]. Thus, timely recognition, management, and prevention of STIs are critical for prevention of HIV acquisition.

Although superior in terms of reliability, laboratory diagnosis of STIs is time-consuming, cost-prohibitive, and requires technology and capacity, which makes its routine use difficult in resource-limited countries. Most of these countries have a high burden of STIs; however, they lack the technical expertise, specialist physicians, and laboratory setup for the diagnosis of these STIs [9]. Furthermore, in situations where laboratory capacity exist, testing may be outsourced to regional facilities and obtaining test results may take up to several weeks. By contrast, syndromic case management algorithms provide an immediate result, allowing for on-site counseling and point-of-care treatment. Furthermore, syndromic diagnosis is feasible and economical in resource-limited countries; it costs less than a fifth of the cost of laboratory-based testing [10]. In 2001, the WHO introduced an updated algorithm for syndromic case management that uses decision trees for the most common signs and symptoms of STIs [11]. Based on the patient's symptoms and gender, different decision-tree diagrams are used. However, these symptoms may be subjective, variable among patients, and a patient with an STI may not manifest overt symptoms. Thus, syndromic diagnosis may miss individuals with asymptomatic STIs [12].

Syndromic diagnosis of STIs is popular in most healthcare systems in sub-Saharan Africa. Nevertheless, ongoing discussions regarding its effectiveness have persisted for years [13]. Several studies have investigated the utility of the syndromic method, especially focusing on target populations such as young women and sex workers [13–15]. Most of these studies were conducted in STI clinics or among particular groups (such as female sex workers), thereby introducing potential biases from convenient sampling. Thus, the findings from these studies may not be generalized to the other populations and clinical settings. This emphasized the need for population-based studies in sub-Saharan Africa to test for the validity of the syndromic approach versus laboratory-based testing for STIs.

The prevalence of transactional sex in sub-Saharan Africa is high, with men being the perpetuators. Adolescent girls and young women who engage in informal sexual exchange for housing, money, and education are at an increased risk of acquiring STIs from men [16]. Wamoyi et al. found that transactional sex is associated with acquisition of HIV; adolescent girls and young women who engage in transactional sex in sub-Saharan Africa are 50% more likely to be infected with HIV, while the findings for men remain inconclusive [17]. Moreover, sub-Saharan African men have higher AIDS-related death, lower awareness, and treatment coverage of HIV compared with women from the region [2, 18]. Despite these statistics, there are few studies from the region focusing on STIs in men. Thus, more studies are needed to elucidate the relationship between STI symptoms and tests among men from the region. Our study addresses this need by evaluating the diagnostic accuracy of the commonly used syndromic approach of STIs in men in the Moshi urban district of Tanzania.

## 2. Methods

### 2.1. Study Participants

This study is based on secondary analysis of data collected during the Moshi Infertility Survey. The rationale, organization, and recruitment for the study have been described in detail elsewhere [12, 19]. In brief, the survey was conducted from November 2002 to March 2003 in the Moshi urban district of Tanzania and involved a two-stage sampling. During the first stage of sampling, 150 clusters of households were selected. In the second stage of sampling, 18 households were randomly selected to participate in the survey from each of the 150 clusters. A total of 2,019 women who were residents of the selected households and 794 men who were listed as husbands or partners of the women were interviewed. Information was collected on sociodemographic characteristics, STIs symptoms, and high-risk sexual behaviors. Blood samples were drawn to test for HIV-1, herpes simplex virus type 2 (HSV-2), and syphilis. Urine samples were obtained to test for chlamydia, gonorrhea, mycoplasma, and trichomonas STIs. The study protocol was approved by the Ethics Committees of the Kilimanjaro Christian Medical Centre, the Tanzania National Institute for Medical Research, the Institutional Review Boards of the Harvard Chan School of Public Health, the University of Maryland, and the Centers for Disease Control and Prevention (CDC), Atlanta, Georgia.

### 2.2. Study Variables

#### 2.2.1. Symptoms of STIs

STI symptoms included at least one of the following: abdominal pain, genital discharge, foul smell in the genital area, excessive genital secretions, swellings in the genital area, itching in the genital area, burning pain on micturition, pain during intercourse, and genital ulcers.

#### 2.2.2. Laboratory Tests of STIs

Blood samples were collected for HIV-1, HSV-2, and syphilis testing. HIV-1 infection was determined by using two enzyme-linked immunosorbent assays (ELISAs). Indeterminate results were resolved by Western blot (Genetic Systems HIV-1 Western blot; Bio-Rad Laboratories, Redmond, WA). Antibodies to HSV-2 were detected using type-specific HSV-2 enzyme immune assay (EIA) (HerpeSelect 2 ELISA, Focus Technologies, Cypress, CA) according to the manufacturer's instructions. Active or recent syphilis was diagnosed if the serum was reactive to both the rapid plasma reagin card test (Macro-Vue; Becton-Dickinson, Cockysville, MD) and the *Treponema pallidum* hemagglutination assay (TPHA) (Wellcosyph HA; Murex Biotech Ltd., U.K.). A positive TPHA test alone was interpreted as evidence of a past infection.

Urine samples were tested for *Chlamydia trachomatis*, *Neisseria gonorrhoeae*, *Trichomonas vaginalis*, and *Mycoplasma genitalium* by using a real-time multiplex polymerase chain reaction (M-PRC) assay with primers and probe sets previously published [19]. In brief, all urine specimens were pooled in groups of 3, and DNA was extracted using the Qiagen Viral RNA kit (Qiagen), which is recommended for extraction of DNA from urine specimens. DNA was then extracted from all specimens falling within positive pools and tested by the M-PCR assay. Sequence-specific detection of M-PCR amplification products was based on TaqMan technology and was performed using the Rotor-Gene 3,000 instrument (Corbett Research, Australia). M-PCR amplifications were performed in 50-*µ*L reaction tubes using 25 *µ*L of sample DNA. A final concentration of 1X PCR Gold buffer (Applied Biosystems), 4 mmol/L MgCl_2_, and 200 *µ*M each of dATP, dGTP, dCTP, and dUTP were used. Two units of AmpliTaq Gold DNA polymerase (Applied Biosystems) and one unit of uracil-N-glycosylase (UNG; Applied Biosystems) were used per 50-*µ*L reaction. Amplification was performed using the following parameters: 95°C for 10 minutes and 50°C for 2 minutes, followed by 50 cycles at 95°C for 20 seconds and 60°C for 60 seconds. Positive controls were prepared from cultures using the Qiagen (Valencia, CA) mini-DNA kit for *N. gonorrhoeae*, *C. trachomatis*, and *T. vaginalis*. *M. genitalium*-positive DNA control was obtained from the ATCC (catalog no. 33530D). The real-time M-PCR assay allows for detection over a broad range of target concentrations with analytical sensitivities of one to 10 genomic copies for *M. genitalium* and *N. gonorrhoeae*, 0.01 to 0.1 genomic copies for *T. vaginalis*, and the equivalent of 0.01 to 0.1 inclusion-forming unit for *C. trachomatis*.

### 2.3. Statistical Analysis

Statistical graphs and descriptive measures (such as mean, median, standard deviation, interquartile range, frequencies, and percentages) were used to summarize the data. Sensitivity and specificity together with their corresponding 95% confidence intervals were calculated to assess the predictive accuracy of each STIs symptom. Kendall's tau-b was used to measure agreements between patient-reported STIs symptoms and laboratory-confirmed STIs. The area under the ROC curve (AUROC) was used to evaluate the overall diagnostic accuracy of the syndromic diagnosis of STIs. The analysis was conducted using the R programming language.

## 3. Results

This study assessed the effectiveness of syndromic versus laboratory testing of STIs among 794 men randomly selected from Moshi district of Tanzania. The average age of the men was 37 years, with a standard deviation of 8.9 years. Forty-four percent (44%) of the participants were in the 31- to 40-year age-group. Only one-third of the participants had a secondary or higher education. Fifty-two percent (52%) worked as either skilled or unskilled laborers, while 20% were unemployed. Most of the men (60%) had their first sex before their 20^th^ birthday; only 16% of them had their first sexual intercourse at marriage or with a cohabiting partner. Most of the participants were in a monogamous relationship for the last 12 months (88%) or 3 years (78%). Moreover, 80% of the participants did not use condom during sexual intercourse in the previous 12 months.

**Figure 1:**
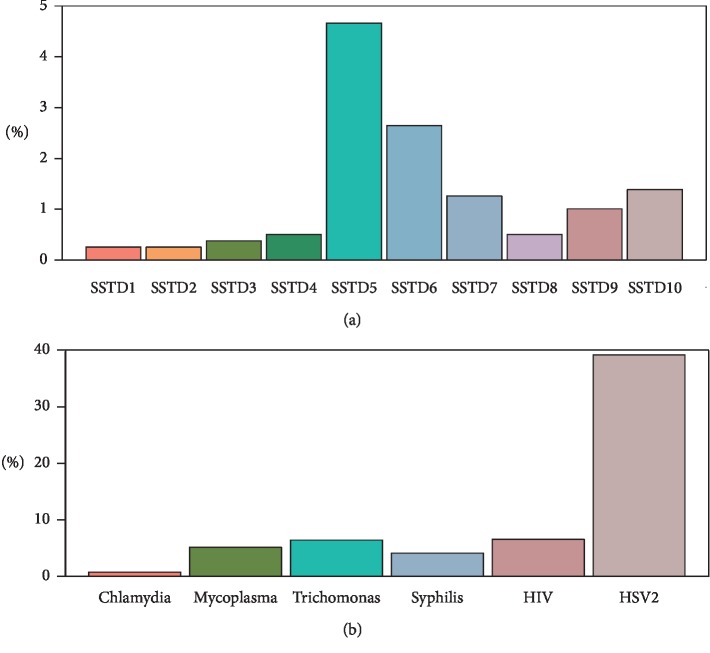
Prevalence of self-reported STIs symptoms and laboratory-confirmed STIs (SSTD1: lower abdominal pain, SSTD2: abnormal genital discharge, SSTD3: foul smell in the genital area, SSTD4: excessive genital secretions, SSTD5: swelling of lymph nodes in the genital area, SSTD6: itching in the genital area, SSTD7: burning pain on micturition, SSTD8: pain during intercourse, SSTD9: genital ulcers and open sores, and SSTD10: other unclassified symptoms).

**Figure 2:**
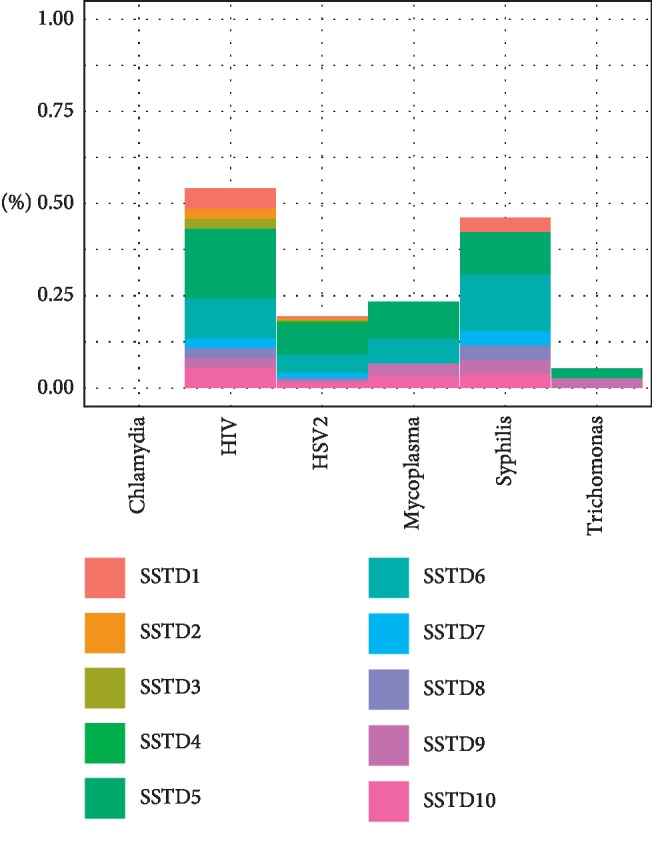
Prevalence of self-reported STIs symptoms among men tested positive for STIs (SSTD1: abdominal pain, SSTD2: abnormal genital discharge, SSTD3: foul smell in the genital area, SSTD4: excessive genital secretions, SSTD5: swelling of lymph nodes in the genital area, SSTD6: itching in the genital area, SSTD7: burning pain on micturition, SSTD8: pain during intercourse, SSTD9: genital ulcers, and SSTD10: other unclassified symptoms).

**Figure 3:**
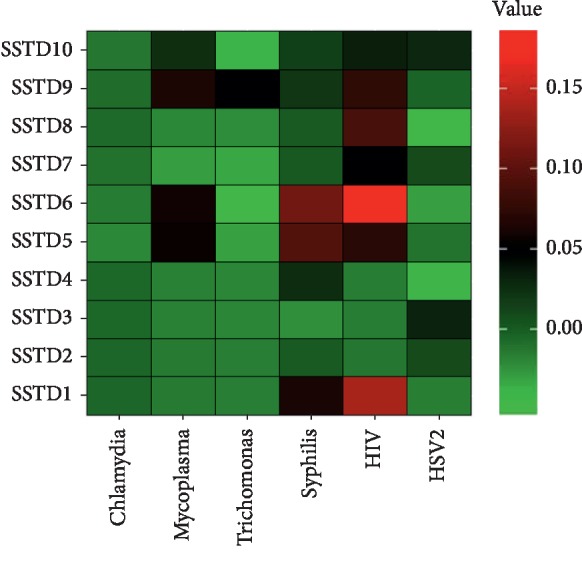
Heat map of Kendall's tau-correlation coefficients between STIs symptoms and laboratory-confirmed STIs (SSTD1: lower abdominal pain, SSTD2: abnormal genital discharge, SSTD3: foul smell in the genital area, SSTD4: excessive genital secretions, SSTD5: swelling of lymph nodes in the genital area, SSTD6: itching in the genital area, SSTD7: burning pain on micturition, SSTD8: pain during intercourse, SSTD9: genital ulcers and open sores, and SSTD10: other unclassified symptoms).

Among the study participants, only 7.9% reported any symptoms, while 46% tested positive for at least one STI, suggesting that many of the men with STIs have asymptotic infections.  Figure 1 shows the rates of STIs symptoms and tests among the men included in the analysis. The highest infection rate was for HSV-2 (39%), followed by HIV-1 (7%), trichomonas (6%), and mycoplasma (5%). The prevalence of other STIs such as syphilis and chlamydia was below 5%. None of the participants tested positive for gonorrhea. The most prevalent STIs symptom was swelling in the genital area (4.7%), followed by itching in the genital area (2.7%), burning pain on micturition (1.3%), genital ulcers or sores (1.0%), excessive genital secretions (0.5%), pain during sex (0.5%), smell from the genital area (0.4%), lower abdominal pain (0.3%), abnormal genital discharge (0.4%), and other nonspecific symptoms (1.4%). The prevalence of STIs symptoms among men tested positive for each STI is presented in Figure 2. Multiple STI symptoms were reported among men tested for HIV-1, HSV-2, syphilis, and mycoplasma. Men who tested positive for chlamydia were asymptomatic, and those who were positive for trichomonas on the average reported two symptoms. Swelling of lymph nodes in the genital area was a common symptom of STIs.  Figure 3 displays a heat map of the correlation coefficients between laboratory-confirmed STI test results and patient-reported STI symptoms. The coefficients were relatively low (range = −0.05–0.24), suggesting no strong associations between STI symptoms and the laboratory-confirmed STI test results.

**Figure 4:**
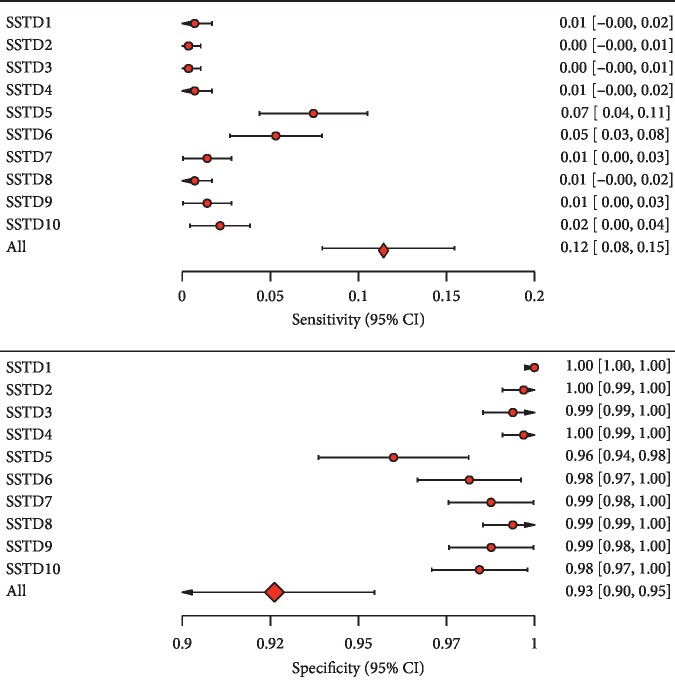
Sensitivities and specificities of STIs symptoms together with their 95% exact confidence intervals (SSTD1: lower abdominal pain, SSTD2: abnormal genital discharge, SSTD3: foul smell in the genital area, SSTD4: excessive genital secretions, SSTD5: swelling of lymph nodes in the genital area, SSTD6: itching in the genital area, SSTD7: burning pain on micturition, SSTD8: pain during intercourse, SSTD9: genital ulcers and open sores, and SSTD10: other unclassified symptoms).

Figure 4 illustrates the sensitivity (true-positive rate) and specificity (true-negative rate) of each STI symptom and the combined symptoms. For each of the STI symptoms, sensitivity tended to be very low, with the maximum being 7.4% (swelling of lymph nodes in the genital area) and the minimum being 0.4% (abnormal genital discharge and smelling in the genital area). Specificities were all above 95%, ranging from 96% to 100%. The combined STI symptoms, with a positive response representing having at least one STI symptom, also had a low sensitivity 0.112 (95% CI: 0.074–0.15) and a high specificity of 0.925 (95% CI: 0.892–0.949). These results suggest that the combined STI symptoms correctly identified 11 of 100 men with laboratory-confirmed STIs and resulted in an 89% false-negative rate. Similarly, the combined STI symptoms correctly identified 92 of 100 healthy men and resulted in an 8% false-positive rate. The reported numbers of STI symptoms were summed to obtain the total number of STI symptoms for each participant. The receiver-operating characteristic (ROC) curve was then constructed using sensitivity and specificity obtained from every possible symptom threshold. The area under the receiver-operating curve (AUROC) was 0.528 (95% CI: 0.505–0.550), indicating that the syndromic approach would correctly identify an infected male patient from a healthy one only 52.8% of the time. Thus, the STI symptoms are no better than a random classifier which has an AUROC of 0.50.

## 4. Discussion

This study investigated the diagnostic accuracy of syndromic diagnosis of STIs relative to laboratory-based diagnosis among men in Moshi district of Tanzania. The study cohort included men who were enrolled in a community-based survey. Forty-six percent of the men tested positive for at least one STI, while only 7.9% reported at least one STI symptom. The most prevalent STI among the study participants was HSV-2 (around 39%), followed by HIV (7%), trichomonas (6%), and mycoplasma (5%). The prevalence rates of other STIs were syphilis (4%) and chlamydia (1%). The high prevalence of HSV-2 may be partly due to the fact that the antibody test could not distinguish between acute and past infections. The prevalence of STI symptoms was below 5%, with the most prevalent STI symptoms being swelling of the lymph nodes in the genital area (4.7%) and itching in the genital area (2.7%). We found little agreement between patient-reported symptoms and laboratory test results for all the STIs considered in the study. Interestingly, itching in the genital area and swelling of lymph nodes in the genital area were associated with STIs compared with the other symptoms we considered.

To evaluate the performance of syndromic diagnosis of STIs, we calculated the sensitivity and specificity for each individual symptom and the combined STI symptoms. For each of the individual STI symptoms, sensitivity was low (ranged from 0.4% to 7.4%), while specificity was relatively high (ranged from 96% to 100%). In this context, the low sensitivity suggests that there is a low probability that a syndromic test will correctly detect an STI in a participant (i.e., there is a high false-negative rate). On the other hand, the high specificity observed in this population-based study suggests a high true-negative rate. The combined STI symptoms, which measure whether a participant has at least one STI symptom, also have very low sensitivity and high specificity. The area under the ROC curve was 0.528, suggesting that the syndromic method can only correctly explain 52.8% of STIs in study participants. Almost half of the time, this approach gives an inappropriate diagnosis.

Studies published in the last decade have yielded conflicting results regarding the effectiveness of syndromic diagnosis of STIs. A study in Delhi by Choudhry et al. found the sensitivity of genital discharge syndrome (GDS) was high for *Neisseria gonorrhoeae* and *Chlamydia trachomatis* (96% and 91%, respectively), whereas the specificity for these STIs was relatively lower (76% and 72%, respectively) [20]. They also found relatively low sensitivity and high specificity of genital ulcer syndrome for herpes simplex virus-2 (HSV-2). Another study by Grijsen et al. indicated that sensitivity tends to be low and specificity high for asymptomatic infection; 67% of urethritis cases in men and 59% of cervicitis cases in women were missed using the syndromic approach [21]. Similar results were reported in a study by Theodora et al. with adequate accuracy for urethral discharge and genital ulcer disease syndromes, but the use of syndromic diagnosis was, however, limited by asymptomatic infections such as chlamydia and gonorrhea in females [22]. In our study, there were not gonorrhea cases; this may be due to the population sampling instead of enrolling patients from STI clinics.

Compared with previous studies, our study has advantages in terms of the sample size and comprehensiveness. Unlike many studies on the performance of syndromic versus laboratory-based diagnosis of STIs, we used population-based study and avoided the potential biases associated with sampling specific groups such as STI clinic attendees or sex workers. However, there are still several limitations of our study. The study included only men who gave consent. Incomplete cases could also lead to a biased result if there was a systematic difference between men who gave consent and those who did not. Furthermore, since symptoms are self-reported, some information from participants may remain underreported considering the sensitivity of STIs. Moreover, HSV-2 antibody positivity without concurrent symptoms ascribed to HSV has no clinical utility. The antibody testing might reflect past and resolved HSV-2 infections. Although we did not have HIV viral load testing, since HIV is a chronic nonrelapsing life-long infection, one can use antibody positivity as infectious state.

## 5. Conclusion

The use of syndromic diagnosis of STIs is pervasive in resource-limited countries because of the ease of implementation, accessibility, and low-cost. However, its performance is woefully inadequate, and there is a need to improve its sensitivity with other clinical correlates or gradually adopt laboratory testing for STI management in resource-limited settings.

## Data Availability

The dataset used in the manuscript is available from the corresponding author on reasonable request.
